# The Antigenic Membrane Protein (Amp) of Rice Orange Leaf Phytoplasma Suppresses Host Defenses and Is Involved in Pathogenicity

**DOI:** 10.3390/ijms24054494

**Published:** 2023-02-24

**Authors:** Zhiyi Wang, Xiaorong Yang, Siqi Zhou, Xishan Zhang, Yingzhi Zhu, Biao Chen, Xiuqin Huang, Xin Yang, Guohui Zhou, Tong Zhang

**Affiliations:** 1 Guangdong Province Key Laboratory of Microbial Signals and Disease Control, College of Plant Protection, South China Agricultural University, Guangzhou 510642, China; 2 College of Marine and Biotechnology, Guangxi Minzu University, Nanning 530008, China; 3 State Key Laboratory for Conservation and Utilization of Subtropical Agro-Bioresources, South China Agricultural University, Guangzhou 510642, China

**Keywords:** phytoplasma, antigenic membrane protein, insect vector, pathogen-host interaction, HR response

## Abstract

Phytoplasmas are uncultivable, phloem-limited, phytopathogenic bacteria that represent a major threat to agriculture worldwide. Phytoplasma membrane proteins are in direct contact with hosts and presumably play a crucial role in phytoplasma spread within the plant as well as by the insect vector. Three highly abundant types of immunodominant membrane proteins (IDP) have been identified within the phytoplasmas: immunodominant membrane protein (Imp), immunodominant membrane protein A (IdpA), and antigenic membrane protein (Amp). Although recent results indicate that Amp is involved in host specificity by interacting with host proteins such as actin, little is known about the pathogenicity of IDP in plants. In this study, we identified an antigenic membrane protein (Amp) of rice orange leaf phytoplasma (ROLP), which interacts with the actin of its vector. In addition, we generated Amp-transgenic lines of rice and expressed Amp in tobacco leaves by the potato virus X (PVX) expression system. Our results showed that the Amp of ROLP can induce the accumulation of ROLP and PVX in rice and tobacco plants, respectively. Although several studies have reported interactions between major phytoplasma antigenic membrane protein (Amp) and insect vector proteins, this example demonstrates that Amp protein can not only interact with the actin protein of its insect vector but can also directly inhibit host defense responses to promote the infection. The function of ROLP Amp provides new insights into the phytoplasma-host interaction.

## 1. Introduction

Phytoplasmas are wall-less bacteria that are members of the class Mollicutes and cause important insect-transmitted diseases in a diverse variety of crops worldwide [1]. These pathogens are restricted to the plant phloem and cause growth disorders, leaf and floral alterations, and abnormal proliferation, sometimes leading to plant death [2]. Plant pathogens, including phytoplasmas, typically employ a range of effectors to modulate the defense and developmental processes of the host plant to benefit their infection [3]. As phytoplasmas inhabit the cytoplasm of the immature and mature sieve cells that constitute the phloem, these bacteria secrete effectors directly into the host cytoplasm of sieve cells via the Sec (secretion pathway)-dependent protein translocation pathway and target other plant cells by symplastic transport [2,3,4]. SecA-secreted proteins are candidate effectors and can be identified by the presence of a signal peptide [2,5] that is cleaved to yield a mature protein during export [6]. Since phytoplasmas are unculturable bacterial pathogens, it is difficult to characterize infection mechanisms at the molecular level [5]. Recently, a couple of phytoplasma effectors have been functionally characterized, and most of them play a crucial role in symptom development and host defense responses [4,7,8,9].

Phytoplasmas are transmitted by a narrow range of phloem-feeding insect species, mainly including leafhoppers, planthoppers, and psyllids, whereas their plant host range is usually broader [10]. Insect vector specificity plays a key role in the epidemiology of several vector-borne pathogens [11,12]. A class of membrane proteins in phytoplasmas have been identified as immunodominant membrane proteins (IDPs), which can directly affect vector insects and host plants and play a crucial role in plant and insect vector transmission [2,13]. Based on chromosomal gene organization and membrane anchor structure, IDPs derived from several phytoplasmas have been classified into three types: immunodominant membrane protein (Imp), immunodominant membrane protein A (IdpA), and antigenic membrane protein (Amp) [14,15]. Imp has a hydrophobic region at the N-terminus as the transmembrane domain and a hydrophilic region at the C-terminus outside the cell [7,16,17]. Imp of *Candidatus* Phytoplasma mali was reported to interact and colocalize with actin in plant cells, indicating its role in the movement of the phytoplasma in host plants [18]. IdpA proteins have an extracellular hydrophilic region in the middle and two hydrophobic regions as the transmembrane domains at both the C-terminus and N-terminus [19,20]. However, the interaction between IdpA and host factors has been less reported. Amp proteins also have a hydrophilic region in the middle, which is located outside the cell; a C-terminal hydrophobic region as the transmembrane domain, which anchors the Amp protein to the cell membrane of the phytoplasma; and an N-terminal hydrophobic signal peptide region which is cleaved during protein procession and translocation [6,21]. However, besides the majority of IDPs, there are several immunogenic membrane proteins present at the surfaces of the phytoplasmas, such as the variable membrane protein, A (VmpA) [22,23]. VmpA proteins possess a putative signal peptide and a potential C-terminal transmembrane domain, and are likely to be anchored in the phytoplasma membrane with a large N-terminal hydrophilic part exposed to the phytoplasma cell surface [24]. VmpA of flavescence dorée (FD) phytoplasma specifically interacted with *Euscelidius variegatus* insect cells in culture and promoted the retention of VmpA-coated beads to the midgut of *E. variegatus* [22].

To date, only a few biological functions of Amp have been studied. Amp of *Candidatus* Phytoplasma asteris, onion yellows strain (OY), has been reported to interact with the microfilament complexes of its vector leafhopper but not with non-vector leafhoppers [25]. Amp of Chrysanthemum yellow phytoplasma (CYP) was also found to interact with the ATP synthase and actin of its vector, but not with the homologous proteins of non-vectors [26,27]. These findings suggest that the complex interaction network between Amp and the proteins of insects determines the vector specificity of phytoplasmas. However, we know little about the role of Amp in regulating host plant gene expression.

Rice orange leaf phytoplasma (ROLP), a member of the “*Candidatus* Phytoplasma asteris” 16SrI-B subgroup, is mainly transmitted by the leafhoppers, *Recilia dorsalis* and *Nephotettix cinticeps* [28]. Rice plants infected with ROLP show yellow and orange streaks appearing from the leaf apex, followed by leaf orange and leaf scorch and, sometimes, even the death of whole plants. Rice orange leaf disease (ROLD) caused by ROLP has been found in several east Asian countries, including Thailand, India, the Philippines, Malaysia, China, and other Asian countries [28,29,30]. Recently, the genome of ROLP has been sequenced, and it is predicted to encode 647 proteins [31]. A gene encoding the Imp of ROLP has been cloned and sequenced, and, using Imp-specific antibodies, researchers have clarified the infection characteristics of ROLP [31,32].

In this study, we used a combination of genome-wide bioinformatics and subsequent functional analyses of the ROLP-encoded proteins to describe an IDP in rice orange leaf phytoplasma, which has been identified as a potential Amp based on protein structure prediction. Protein interaction assay showed that it can interact with the actin protein of its vector leafhopper and confirmed this is an Amp protein. Although Amp was shown to bind to host proteins and could be essential for phytoplasma transmission by insect vectors, its function in plants has not yet been described. Because phytoplasmas propagate in both insect and plant hosts, the study of the function of phytoplasma proteins expressed in plants is required. In this study, we generated transgenic rice plants expressing the Amp, and the protein was also transiently expressed in *Nicotiana benthamiana* by the potato virus X (PVX) system. We found that ROLP Amp can enhance the proliferation of ROLP and PVX and cause severe symptoms in rice and *N. benthamiana* plants, respectively. In addition, we proved that ROLP Amp can inhibit defense responses in rice plants. These data first suggested that ROLP Amp is responsible for phytoplasma pathogenicity in plants and suppressing host defense responses.

## 2. Results

### 2.1. Identification of Amp Encoded by ROLP

**Figure 1:**
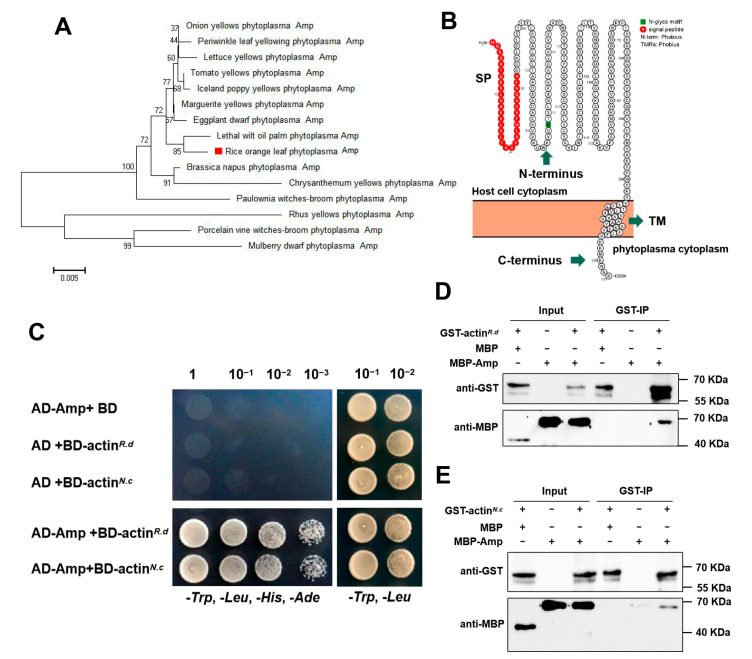
Identification of ROLP-Amp. (**A**) Phylogenetic tree of Amp orthologous to different phytoplasmas. The phylogenetic tree was constructed by Mega 5.0, bootstrap values (>50) are indicated in each node. The red square indicates the Amp encoded by ROLP. (**B**) Prediction features of ROLP Amp. (**C**) Yeast two-hybrid assays were conducted to confirm the interaction between ROLP-Amp and vector actin. Yeast strain Y2HGold cells co-transformed with the indicated plasmids were cultured separately on the SD-Trp-Leu-His-Ade and SD-Trp-Leu selection medium. (**D**,**E**) GST pull-down assays showing Amp-actin (*R. dorsalis*) (**D**) and Amp-actin (*N. cincticeps*) (**E**) interactions in vitro, respectively. GST-tagged actin (*R. dorsalis*) or GST-tagged actin (*N. cincticeps*) was incubated with MBP-tagged Amp or MBP and immunoprecipitated with glutathione-Sepharose beads. The pull-down and input proteins were detected by western blotting assays with anti-MBP and anti-GST antibodies.

Phytoplasmas are wall-less pathogens; therefore, their membrane proteins can directly contact the cells of their host plant or vector. Among these proteins, Amp is thought to play an important role in the interaction between host plants and vector insects. To identify the Amp of ROLP, we screened the whole genome sequence of ROLP and compared it with the sequences of Amp genes from other phytoplasmas. Through the comparison, a protein encoded by ROLP (NCBI accession number: WP071345415.1) with high homology with Amp from other phytoplasmas was found. The phylogenetic tree further revealed that the Amp of ROLP shared 98.6% and 95.5% sequence similarity to the Amp of OY-M and CYP, respectively (Figure 1A). Furthermore, we predicted the structure of the ROLP Amp using Protter (http://wlab.ethz.ch/protter/ (accessed on 25 April 2022)). Results revealed that it has an N-terminal hydrophobic signal peptide region and a C-terminal hydrophobic region as the transmembrane domain, which is the typical Amp protein structure (Figure 1B and Figure S1).

A previous study has suggested that the Amp may be involved in the specific recognition of phytoplasma by its vectors [25]. To investigate whether ROLP-encoded potential Amp has a similar function, we used a yeast two-hybrid (Y2H) assay to identify the interaction between ROLP-encoded Amp and its vector insect-encoded actin. The results showed that ROLP-encoded Amp interacts with the actin of its vectors, *R. dorsalis* and *N. cincticeps* (Figure 1C). The interaction between Amp and the actin of vectors was further confirmed by the GST pull-down assay (Figure 1D, E). The results showed that we have identified an Amp of ROLP.

### 2.2. Expression Characteristics of ROLP Encoded Amp in Rice and R. dorsalis

**Figure 2:**
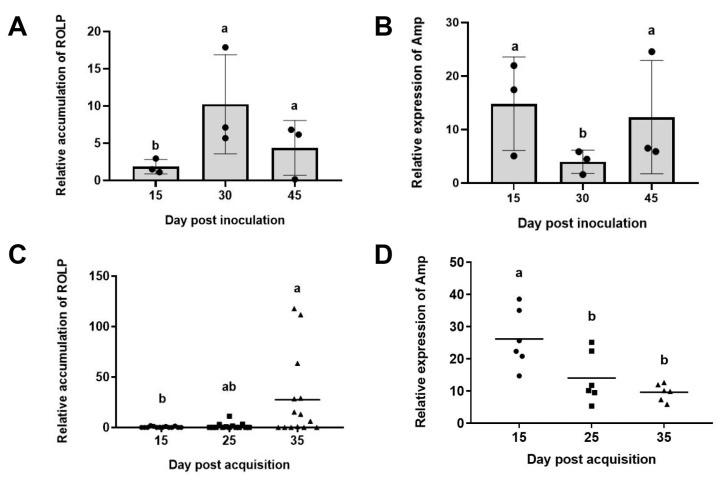
Expression characteristics of ROLP encoded Amp in rice plants and *R. dorsalis* (**A**) Relative accumulation of ROLP in rice plants. Total DNAs were extracted from mock-infected or ROLP-infected rice leaves at 15, 30, and 45 dpi. Values represent the mean of three biological repeats normalized with *OsEF1α* as an internal reference. (**B**) Relative expression of *Amp* in ROLP-infected rice plants. Total RNAs were extracted from mock-infected or ROLP-infected rice leaves at 15, 30, and 45 dpi. Values represent the mean of three biological repeats normalized with ROLP *NusA* as an internal reference. (**C**) Relative accumulation of ROLP in *R. dorsalis* leafhoppers. Total DNAs were extracted from individual leafhoppers at 15, 30, and 45 dpa. Values represent the mean of fourteen biological repeats normalized with leafhopper *Actin* as an internal reference. (**D**) Relative expression of *Amp* in ROLP-infected *R. dorsalis*. Total RNAs were extracted from individual leafhoppers at 15, 30, and 45 dpa. Values represent the mean of six biological repeats normalized with ROLP *NusA* as an internal reference. Each point in the chart indicates a biological repeat, and different letters indicate significant difference (*p* < 0.05) based on the Tukey–Kramer HSD test. The experiments were repeated three times with similar results.

We first investigated ROLP accumulation in rice plants and *R. dorsalis.* Results showed that the accumulation of ROLP increased from 15 to 30 days post inoculation (dpi) and reduced at 45 dpi (Figure 2A) in rice plants. We then used the phytoplasma conserved *NusA* gene as internal controls, and the expression level of Amp was investigated. The results showed that the expression level of *Amp* was relatively higher at 15 dpi and 45 dpi, and lower at 30 dpi (Figure 2B).

Similarly, we investigated ROLP accumulation in *R. dorsalis*. Results showed that the accumulation of ROLP gradually increased from 15 to 35 days post-acquisition (dpa) (Figure 2C), while the expression of *Amp* gradually decreased (Figure 2D). These results indicated that *Amp* expression was opposite to ROLP accumulation in the host and vector, suggesting that Amp plays an important role in promoting the infection of ROLP in the early stage.

### 2.3. Amp Promotes the Proliferation of ROLP in Rice Plants

**Figure 3:**
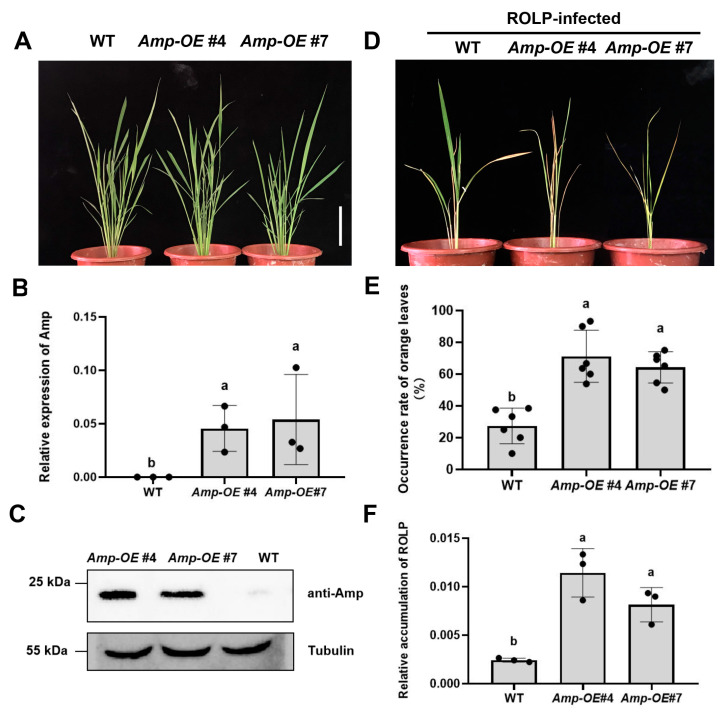
ROLP-encoded Amp promotes the propagation of ROLP in rice plants. (**A**) Phenotypes of WT (NIP) and Amp-overexpressing (*Amp-OE*) plants. Transgenic overexpressing ROLP-Amp lines (#4 and #7) showed no apparent differences from WT plants. Scale bar = 10 cm (**B**) The expression of Amp in transgenic plants was examined by RT-qPCR. Total RNAs were extracted from WT and *Amp-OE* plants. Values represent the mean of three biological repeats normalized with *OsEF1α* as an internal reference. (**C**) Western blot analysis of Amp protein accumulation in WT and *Amp-OE* plants. Total proteins were extracted from the rice leaves and were detected by anti-Amp polyclonal antibody. Immunoblot detection of tubulin was used as loading control. (**D**) Phenotypes of ROLP-infected WT and *Amp-OE* plants. Photos were taken at 4 weeks after ROLP-inoculation. (**E**) The percentages of orange leaves in ROLP-infected WT and *Amp-OE* plants. The orange leaves and total leaves of individual plants were counted, and the values represent the mean of six biological repeats. (**F**) Relative accumulation of ROLP in inoculated WT and *Amp-OE* plants. Total DNAs were extracted from ROLP-infected rice leaves at 15 dpi. Values represent the mean of three biological repeats normalized with *OsEF1α* as an internal reference. For (**B**,**E**,**F**), each point in the chart indicates a biological repeat, and different letters indicate significant difference (*p* < 0.05) based on the Tukey–Kramer HSD test. The experiments were repeated three times with similar results.

To investigate the role of Amp in ROLP infection, we generated *Amp*-overexpression (*Amp-OE*) transgenic rice plants, which constitutively express ROLP Amp without its signal peptide but fused with a 4 × Myc tag on its N-terminus (Figure S2). The *Amp-OE* transgenic lines showed a normal growth phenotype compared with wild type (WT) (Figure 3A), and the expression of *Amp* in the transgenic lines was confirmed by RT-qPCR (Figure 3B) and Western blot (Figure 3C).

Then, we inoculated the WT and *Amp-OE* plants with ROLP by leafhopper inoculation. We found that, at four weeks post-inoculation (wpi), the *Amp-OE* lines exhibited much more severe disease symptoms (Figure 3D). The ROLP-infected *Amp-OE* plants had more orange leaves compared to the WT plants (Figure 3E). Consistent with the observed phenotypes, the accumulation of ROLP was higher in *Amp-OE* rice plants than in WT plants (Figure 3F). These results convincingly demonstrate that Amp plays a positive role in ROLP infection.

### 2.4. Amp Suppressed Host Defense Responses through SA and Ethylene Biosynthesis

**Figure 4:**
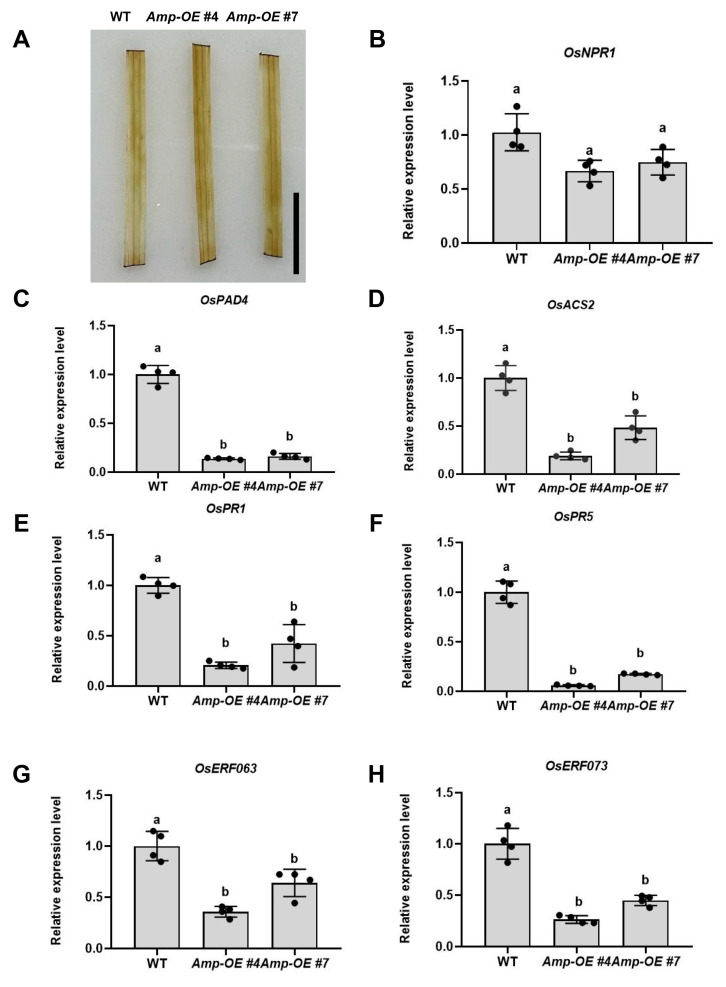
Amp suppressed host defense responses through SA and ethylene biosynthesis. (**A**) Assessment of H_2_O_2_ accumulation by DAB staining. DAB-treated leaves of WT and *Amp-OE* were observed 12 h post-infiltration. Scale bar = 1 cm. (**B**–**H**) Relative expression of *OsNPR1* (**B**), *OsPAD4* (**C**), *OsACS2* (**D**), *OsPR1* (**E**), *OsPR5* (**F**), *OsERF063* (**G**), and *OsERF073* (**H**) in WT and *Amp-OE* plants, respectively. Total RNAs were extracted from WT and *Amp-OE* plants. Values represent the mean of four biological repeats normalized with *OsEF1α* as an internal reference. Each point in the chart indicates a biological repeat, and different letters indicate significant difference (*p* < 0.05) based on the Tukey–Kramer HSD test. The experiments were repeated three times with similar results.

Since Amp can promote the infection of ROLP in rice plants, we intended to test whether Amp was a functional effector involved in plant immunity. Hypersensitive response (HR) is commonly used as an indicator for effector-triggered immunity (ETI), and HR accompanies H_2_O_2_ accumulation [33]. We investigated whether the Amp could induce H_2_O_2_ accumulation in rice plants. DAB staining showed that the deep brown color was observed neither in the leaves of *Amp-OE* plants nor in WT plants (Figure 4A), which indicates that Amp does not induce H_2_O_2_ accumulation or trigger the immunity defense through HR.

Then, we examined the expression of ETI and PAMP-triggered immunity (PTI)-related genes in *Amp-OE* and WT plants. Firstly, we performed qRT-PCR analysis of the pathogenesis-related gene *OsNPR1*. Results showed that the expression level of *OsNPR1* was not significantly different between *Amp-OE* and WT plants (Figure 4B). Since many plant pathogens actively manipulate plant defense hormone pathways for pathogenesis, we next investigated the expression of several plant hormone biosynthesis and response-related genes. Results showed that the SA synthesis gene *OsPAD4* [34,35] and the ethylene biosynthesis-related enzyme *OsACS2* [36] were significantly reduced in *Amp-OE* plants than in WT plants (Figure 3C,D). Furthermore, we detected the expression of the SA-regulated genes *OsPR1* and *OsPR5* [37], and the ET downstream genes *OsERF063* and *OsERF073* [38]. Expression of all the genes was significantly reduced in *Amp-OE* plants compared to WT (Figure 3E–H). Together, these results suggest that ROLP-encoded Amp probably suppresses SA and ethylene-mediated disease resistance.

### 2.5. Amp Enhances PVX Virulence in Tobacco

To further verify the pathogenicity of Amp, we then used the PVX vector to express Amp in tobacco plants. The recombinant PVX-Amp was infiltrated into *N. benthamiana* leaves, and leaves infiltrated with PVX without an insert were used as controls. At 12 dpi, the leaves infiltrated with PVX-Amp showed obviously more curl and mosaic than the leaves infiltrated with PVX (Figure 4A,D). RT-PCR results indicated that *Amp* was expressed in the viral progeny (Figure 4B). qRT-PCR results showed that the PVX *CP* transcript level was significantly higher in PVX-Amp-infected plants than in their PVX-infected counterparts (Figure 4C).

To investigate whether the increased accumulation of PVX-CP is accompanied by hypersensitive responses, the accumulation of H_2_O_2_ was examined in a DAB staining assay. The upper, non-infiltrated leaves of PVX- and PVX-Amp-infected plants at 12 dpi were analyzed. The PVX-Amp-infected leaves accumulated higher amounts of H_2_O_2_ than the PVX-infected leaves (Figure 4D). Cell death was also examined by trypan blue staining. The leaves were only lightly stained, with no significant differences between the PVX- and PVX-Amp-infected leaves, indicating that Amp does not induce cell death (Figure 4D). These data suggest that ROLP-encoded Amp can promote the infection of other pathogens and increase the H_2_O_2_ content in tobacco plants.

## 3. Discussion

Arthropod-borne pathogens are transmitted by specific arthropod vectors (mainly insects). As an important type of arthropod-borne pathogen, phytoplasma has shown highly specific interactions with its insect vector. Phytoplasma-encoded Amp is anchored on the membrane of phytoplasma cells and is in direct contact with hosts or vector factors, which are presumably involved in determining vector specificity during the penetration of phytoplasma across gut and salivary gland barriers in the vector [25,27]. For instance, OY phytoplasma-encoded Amp formed a complex with insect microfilaments, including actin, the heavy chain and light chain of myosin, from its vector leafhopper species but not from non-vector species [25]. Similar results were obtained from chrysanthemum yellow (CY) phytoplasma, the Amp of which selectively interacted with actin and the ATP synthase of its vector leafhopper species but not with that of non-vector species [27]. In this study, we identified an Amp of ROLP, and through protein–protein interaction assays, we confirmed the interaction between the ROLP-encoded Amp and the actin from the leafhopper vectors, *R. dorsalis* and *N. cincticeps*, suggesting the interaction might be involved in the vector specificity of ROLP. Further experiments are required to verify the interaction of ROLP Amp with the actin from non-vector and to confirm whether Amp determines the vector specificity of ROLP.

The genome sequence of ROLP has significantly contributed to our understanding of ROLP biology. Studies have shown that differential regulation of phytoplasma gene expression plays an important role in adaptation to various environments encountered within its hosts [39]. The expression levels of OY-M *PAM064* and *PAM695* genes in OY-infected leafhoppers were significantly higher than those in OY-infected plants [40]. In addition, the *PME2* (Protein in Malus Expressed 2) of apple cluster phytoplasma (*Candidatus* Phytoplasma mali, *Ca.*P. mali) is expressed only in the roots and leaves of susceptible apple trees [41]. Moreover, the expression level of AY-WB Amp was 3-fold higher in plants than in vector insects [39]. In this study, we investigated the accumulation of Amp during ROLP infection in *R. dorsalis* and rice plants. Results showed that the expression level of *Amp* was relatively higher at 15 dpi and 45 dpi and lower at 30 dpi. Since Amp is a membrane protein, we assumed that 15 dpi is an early stage of ROLP infection and that a higher expression level of Amp can help ROLP establish a faster infection, whereas at 30 dpi, ROLP mainly replicates and accumulates in the plant cells, so it needs to secrete many more other effectors to conquer plant immunity. At 45 dpi, ROLP has a higher accumulation in infected plants; this is the time for ROLP to transmit, so it secretes more Amp proteins to help ROLP establish infection in insects that are feeding on the sap of infected plants. Although Amp has been shown to bind to insect Actin [25,27], this binding has not been reported to exhibit any negative effect on the life cycle of the vector insect. Therefore, the results of our study support the hypothesis that binding of an immunodominant protein to vector Actin could be beneficial for phytoplasma survival (probably for colonization, infection, and transmission).

**Figure 5:**
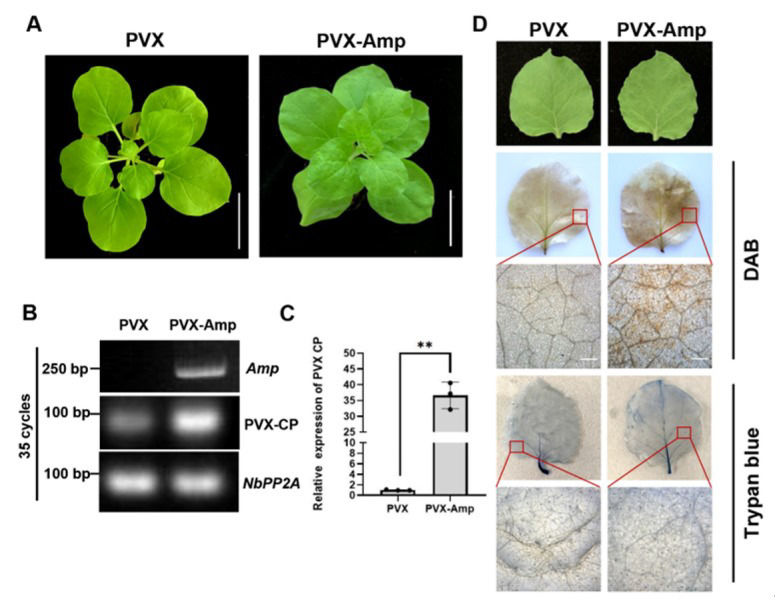
Effect of Amp on potato virus X (PVX) virulence in *N. benthamiana.* (**A**) Phenotypes of PVX and PVX-Amp-infected *N. benthamiana* plants. Photographs were taken at 7 days post-infiltration (dpi). Scale bar = 5 cm. (**B**) Detection of *Amp* and PVX-CP expression in *N. benthamiana* leaves infiltrated with *Agrobacterium tumefaciens* carrying PVX or PVX-Amp through RT-PCR. The expression of *NbPP2A* was used as internal control. (**C**) Relative expression of the PVX CP gene in PVX and PVX-Amp infected *N. benthamiana* plants. Total RNAs were extracted from tobacco leaves. Values represent the mean of three biological repeats normalized with *NbPP2A* as an internal reference. Each point in the chart indicates a biological repeat. Student’s *t*-test was used for analyses (** *p* < 0.01). (**D**) PVX-Amp-infected tobacco leaves exhibit hypersensitive response characteristics. Brown insoluble polymer from DAB staining indicates H_2_O_2_ accumulation. Trypan blue staining indicates the cell death resulting from each treatment. The scale bar for whole leaves = 2 cm, and the scale bar for enlarged area = 500 µm.

Amp has played an important role in the evolution of phytoplasmas, and there is a strong positive selection of Amp in phytoplasmas [14]. Generally, it is believed that pathogen genes that are subject to positive selection play important functions in host immunity and defense responses [3]. In this study, we generated ROLP-encoded Amp transgenic rice plants. Through ROLP infection, we found that the Amp transgenic plants showed more severe symptoms and accumulated higher ROLP titers than WT plants, indicating that Amp may promote ROLP infection in rice plants. The effectors of pathogenic microbes often interfere with plant defense responses such as pattern-triggered immunity (PTI) and effector-triggered immunity (ETI) [42]. Three well-studied phytoplasma effectors (SAP11, SAP54, and TENGU) have been shown to function mainly in manipulating plant development and/or suppressing plant defense responses against their insect vectors. SAP05 mediates the degradation of multiple developmental regulators through a ubiquitination-independent mechanism, leading to delayed plant aging and simultaneous proliferation of vegetative tissue and shoots [9]. SAP11, secreted by aster yellows phytoplasma strain witches’ broom (AY-WB), can not only induce smaller rosettes, severely crinkled leaves, crinkled siliques, and witches’ broom phenotypes in plants but can also down-regulate the expression of *LOX2* and JA synthesis in SAP11-transgenic plants [43]. TENGU, another witches’ broom-inducing effector belonging to OY-M, can suppress auxin signaling and biosynthesis pathways in *Arabidopsis* [29]. Another AY-WB effector, SAP54, transforms the flowers of *Arabidopsis* into leaf-like vegetative tissues, and plants with a SAP54-induced phenotype are more attractive for colonization by phytoplasma leafhopper vectors [44]. However, this phenomenon was not observed in *N. benthamiana* plants expressing the Imp of *Candidatus* Phytoplasma mali [18], and such IDP pathogenicity has not been investigated yet. In this study, we also found that overexpressing Amp in rice plants did not exert any remarkable change in phenotype compared with the WT plant, suggesting that immunodominant membrane proteins are not involved in growth deformations. To further investigate whether Amp regulates defense responses in plants, we conducted a PVX-based expression assay to determine the pathogenicity of Amp. Our data suggested that ROLP-Amp can enhance PVX pathogenicity by increasing PVX RNA accumulation (Figure 5). We also found that PVX-Amp-infected plants can induce hypersensitive responses, whereas the *Amp-OE* rice plants do not trigger the immunity defense through HR (Figure 4A). Since a higher accumulation of viruses is always accompanied by hypersensitive responses [45], the HR induced in PVX-Amp is probably due to the higher accumulation of PVX and not the Amp itself.

## 4. Materials and Methods

### 4.1. Plant Materials

Rice plants cv. Nipponbare were grown inside a greenhouse maintained at 28–32 °C and 60 ± 5% relative humidity with a 12 h photoperiod. Transgenic rice plants (cv. Nipponbare background) were generated at the Biogle Genome Editing Center, Jiangsu, China. *N. benthamiana* were grown in environmental growth chambers maintained at 23 °C with a 16 h photoperiod, 6000 lux of light intensity, and 65% relative humidity.

### 4.2. Phytoplasma and Insects

ROLP-infected rice plants and leafhopper, *R. dorsalis*, were maintained in our laboratory. To obtain ROLP-infected leafhoppers, ~30 *R. dorsalis*, 3–4 larval nymph stage, were transferred to ROLP-infected rice plants for 35 days. The *R. dorsalis* adults were used for experiments or transferred to rice seedlings (~30 seedlings) to generate new batches of ROLP-infected plants and leafhoppers. Briefly, two-week-old seedlings were exposed to the ROLP-carrying leafhoppers (2–3 insects per plant for 14 days). Fourteen days after inoculation, the insects were removed, and the plants were kept in the same conditions.

For the detection of ROLP in infected rice plants and leafhoppers, total DNA was extracted from the leaves of rice plants or leafhoppers by the cetyl trimethylammonium bromide (CTAB) method [46]. A PCR assay was performed to detect the *FisH*1 gene to verify ROLP-infected rice plants and insects according to our previous description [47].

### 4.3. Generation of Amp Transgenic Lines

The full-length ORF of Amp was cloned into the pENTR/D-TOPO vector (ThermoFisher Scientific, Waltham, MA, USA). The cloned sequences were then transferred into the pBA35S-FlagMyc4 vector (under the control of the cauliflower mosaic virus 35S promoter) [48] using a Gateway LR reaction kit (ThermoFisher Scientific, Waltham, MA, USA) as instructed. The resulting plasmids were transformed into *Agrobacterium tumefaciens* strain GV3101. The bacterial cell suspension was used for the generation of Amp transgenic rice plants (cv. Nipponbare background) as described previously [49]. The T0 transgenic plants were screened by quantitative real-time PCR, and the primers are given in Supplemental Table S1.

### 4.4. Quantitative Real-Time PCR

For the quantification of ROLP accumulation in insects and rice plants, total DNA was extracted using the CTAB method. Three independent samples of ROLP-infected rice plants were tested using *qPCR*. Since gene expression varies widely among individual insects, fourteen independent samples of ROLP-infected insects were tested using *qPCR*. The phytoplasma-conserved *NusA* gene was detected as a target [50], and *OsEF1α* and *Actin* in rice and insects, respectively, were used as internal controls.

For detection of *Amp* expression level in ROLP-infected plants and insects, three independent ROLP-infected rice plants or six independent ROLP-infected insects were randomly chosen for qRT-PCR. To verify that *Amp-OE*#4 and #7 were overexpressed, three independent plants from each line were selected and proceeded for qRT-PCR. For the determination of the expression level of defense-related genes, four independent rice plants from each line were randomly chosen for qRT-PCR. For comparison with the PVX accumulation level, three PVX and PVX-Amp-infected plants were randomly chosen for qRT-PCR.

The total RNA of all the samples was extracted from the leaves of plants or insects with Total RNA Extraction Reagent (Vazyme, Nanjing, China) according to the manufacturer’s instructions. cDNA was synthesized using the isolated total RNA, an oligo (dT) primer, and a reverse transcriptase (Takara, Dalian, China). Quantitative PCR reactions were carried out on a CFX96 Touch real-time PCR detection system (Bio-Rad, Hercules, CA, USA) using the SYBR Premix Ex Taq^TM^ II kit (Takara, Dalian, China). Briefly, 2 µL of template cDNA, 5× SYBR Green, and 10 mM of each primer were mixed together in a total volume of 10 µL, and PCR reactions were run as follows: 10 min at 96 °C, followed by 40 cycles of 60 s at 95 °C, 60 s at 60 °C, and 30 s at 72 °C; 10 min at 72 °C. The *NusA* gene of ROLP, *OsEF1α* of rice, and *NbPP2A* of tobacco were used as internal controls, and the relative expression levels were calculated by the 2^−ΔΔC(t)^ method [51]. Three technical replicates were run for each biological replicate. All the experiments were performed at least three times with similar results, and a representative group of results is displayed. Primers used for qRT–PCR are listed in Supplemental Table S1.

### 4.5. Y2H Assay

The full-length Amp and Actin of different insect species were amplified by PCR with the primers listed in Supplemental Table S1. The amplified products were inserted into the yeast expression vectors, pGADT7 and pGBKT7, to generate the constructs for the Y2H assay. To examine protein–protein interactions, different combinations of pGBK and pGAD plasmids were transformed into yeast strain Y2HGold cells (Weidi, Shanghai, China). The transformants were cultivated on the SD/-Leu/-Trp (SD-L-T) medium and then on the SD/-Leu/-Trp-His-Ade (SD-L-T-H-A) selection medium to determine the protein-protein interaction. Yeast cells were photographed 3 days post-incubation at 30 °C. All the experiments were repeated three times with similar results.

### 4.6. GST Pull-Down Assay

The pull-down assay was performed as previously described with minor modifications [52]. The full-length Amp was amplified and inserted into the pMBP28 vector, and the actins of *R. dorsalis* and *N. cincticeps* were amplified and inserted into the pGEX4T1 vector. The recombinant GST- and MBP-tagged proteins were purified using glutathione Sepharose 4B beads (GE Healthcare, Uppsala, Sweden) and amylose resin (New England Biolabs, Ipswich, MA, USA) as instructed by the manufacturer. Then, 4 μg purified MBP or MBP-Amp was incubated with 2 μg purified GST-actin in 200 µL PBS buffer (10 mM Na_2_HPO_4_, 2 mM NaH_2_PO_4_, 135 mM NaCl, 4.7 mM KCl, pH 7.0), and then incubated with 20 μL glutathione Sepharose 4B beads at 4 °C for 2 h. After five washes with reaction buffer, the resin-bound proteins were boiled in SDS buffer for Western blotting analysis with anti-GST and anti-MBP antibodies.

### 4.7. Western Blot

Total protein was extracted from 0.2 g leaf samples with 200 µL extraction buffer (50 mM Tris-HCl (pH 6.8), 9M urea, 4.5% SDS, and 7.5% β-mercaptoethanol). Samples were centrifuged at 12,000× *g* for 2 min, and the upper liquid phase of each sample was analyzed via electrophoresis on SDS-PAGE gels. The separated proteins were transferred to PVDF membranes (Millipore, Billerica, MA, USA) and detected using antibodies against MBP, GST, Myc, or tubulin (Abmart, Shanghai, China). The detection signal was then visualized using the Immobilon Western Chemiluminescent HRP Substrate as instructed (Millipore, Bedford, MA, USA) and visualized on ChemiDoc XRS+ (Bio-Rad, Hercules, CA, USA). The bands of tubulin were used as the loading control.

### 4.8. PVX Infection Assays

The full-length ORF of Amp was inserted into the pGR107 vector [53] to generate PVX-Amp, which was transformed into *A. tumefaciens* strain GV3101. Cultures of transformed GV3101 cells were grown in LB medium containing rifampicin (50 mg/mL) and kanamycin (50 mg/mL) at 28 °C for 48 h. Transformants were identified based on colony PCR. The cells of a GV3101 culture for PVX or PVX-Amp were resuspended in infiltration medium (5 mg/mL glucose, 10 mM MES, 10 mM MgCl2, and 200 mM acetosyringone) for an optical density at 600 nm (OD600) of 0.6. The Agrobacterium cultures carrying PVX-Amp and PVX infiltrated seven 3-week-old *N. benthamiana* plants, respectively. Total RNA was extracted 7 d after inoculation, and viral RNAs were detected by primers targeting the coat protein (CP) of PVX.

### 4.9. DAB and Trypan Blue Staining Assays

For the DAB and Trypan blue staining experiments, leaves were collected from each of the seven plants infected with PVX or PVX-Amp. To detect the accumulation of H_2_O_2_, the leaves were stained with 3,3′-diaminobenzidine (DAB) solution. Leaves were collected at 7 d post-inoculation (dpi) and infiltrated in 1 mg/mL DAB solution (pH 5.7) for 8 h in darkness. The leaves were discolored by boiling 95% ethanol for 10 min and then analyzed. To detect cell death, the leaves were boiled for 3 min in Trypan blue solution (1 mg/mL Trypan blue in water: glycerol:lactic acid:phenol, 1:1:1:1 *v*/*v*). Then, the leaves were infiltrated overnight in a chloral hydrate solution (250 g chloral hydrate dissolved in 100 mL water). All the stained leaves were observed with a Nikon microscope (A1 HD-25).

### 4.10. Statistical Analyses

Differences were analyzed using a two-way analysis of variance (ANOVA) with Tukey’s honest significant difference (HSD) test for multiple comparisons or a one-way *t*-test for comparisons between two means. A *p*-value ≤ 0.05 was considered statistically significant. All analyses were performed using SPSS version 2.0 (SPSS, Inc. Chicago, IL, USA).

## 5. Conclusions

In summary, we identified the Amp encoded by ROLP and clarified its function as a pathogenicity-related protein. Additionally, the molecular mechanisms of Amp induction or the suppression of host defense responses need to be thoroughly investigated in the future. The data presented here may be useful for elucidating the ROLP infection cycle and may be relevant for the development of improved methods for the prevention and control of this pathogen.

## Data Availability

The data supporting the findings of this study are available from the corresponding author upon reasonable request.

## Supplementary Materials

The following supporting information can be downloaded at: https://www.mdpi.com/article/10.3390/ijms24054494/s1.

## Abbreviations

Amp	Antigenic membrane protein
*A. tumefaciens*	*Agrobacterium tumefaciens*
CP	Coat protein
CYP	Chrysanthemum yellow phytoplasma
DAB	Diaminobenzidine
ET	Ethylene
ETI	Effector-triggered immunity
*E. variegatus*	*Euscelidius variegatus*
HR	Hypersensitive response
IDP	Immunodominant membrane protein
Imp	Immunodominant membrane protein
IdpA	Immunodominant membrane protein A
*N. benthamiana*	*Nicotiana benthamiana*
*N. cincticeps*	*Nephotettix cinticeps*
OY	Onion yellow strain
OY-M	Onion yellow strain, a mildly pathogenic line
PTI	PAMP-triggered immunity
PVX	Potato virus X
ROLP	Rice orange leaf phytoplasma
*R. dorsalis*	*Recilia dorsalis*
SA	Salicylic acid
Sec	Secretion pathway.
