# Supplementary material for: The Antigenic Membrane Protein (Amp) of Rice Orange Leaf Phytoplasma Suppresses Host Defenses and Is Involved in Pathogenicity
Source: Int J Mol Sci. 2023 Feb 24;24(5):4494. doi: 10.3390/ijms24054494 (PMC10003417; doi:10.3390/ijms24054494)
Supplement: Supplementary file 1 [file ijms-24-04494-s001.zip › Figure S1.pdf]

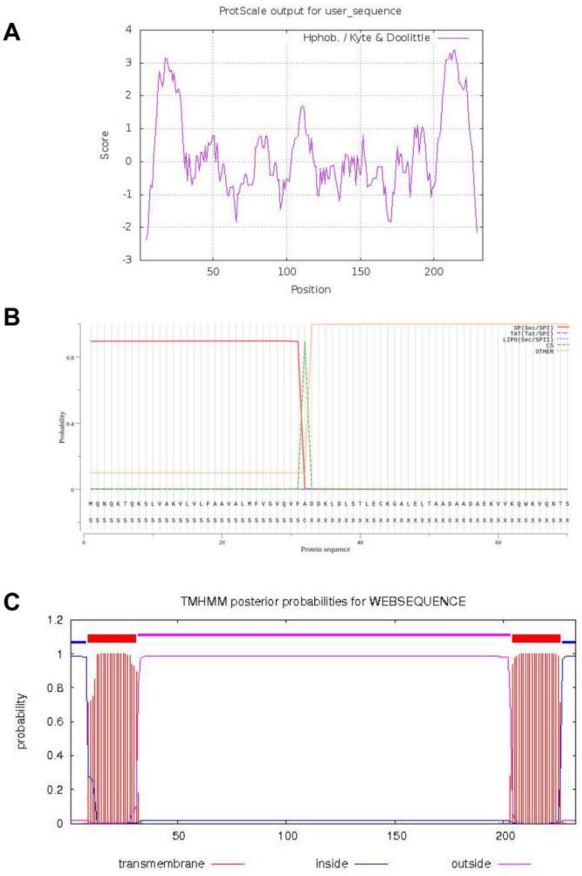

Figure S1: Analysis of ROLP-Amp. (A) Amp Hydrophilic Analysis; (B) Prediction of ROLP-Amp protein signal peptide cleavage site; (C) Prediction of transmembrane domain of ROLP-Amp
