# Supplementary material for: The Antigenic Membrane Protein (Amp) of Rice Orange Leaf Phytoplasma Suppresses Host Defenses and Is Involved in Pathogenicity
Source: Int J Mol Sci. 2023 Feb 24;24(5):4494. doi: 10.3390/ijms24054494 (PMC10003417; doi:10.3390/ijms24054494)
Supplement: Supplementary file 1 [file ijms-24-04494-s001.zip › Table S1.pdf]

Table S1. Primers used in this study

Primer Name	Sequence（5'-3'）
Amp-F	ATGGATGATAAACTAGATTTAAGCAC
Amp-R	TTATTTATTGTTTTTGTTTTTTTTAAC
1355CF	GCAAAAGCACTTGCAGGAGAG
1355CR	GCTTGTC TTGCTTTTATGTCGG
qOsEF1a-F	ACATTGCCGTCAAGTTTGCTG
qOsEF1a-R	ACATTGCCGTCAAGTTTGCTG
Actin-F	CATCAGGGTGTCATGGTGGG
Actin-R	GGTCTCAAACATGATCTGGGTCA
qAmp-F	CCAGCTTCTGCATCTACTTTGC
qAmp-R	GTAGTGGTTGCTGATAATAAAGTAAC
NusA-F	GCATTTCTGGCGAACGTACCA
NusA-R	CAACTTGAAGGAATGAAGCAGG
pGADT7-Amp-F	GCCATGGAGGCCAGTGAATTCGATGATAAACTAGATTTAAGCAC
pGADT7-Amp-R	ACGATTCATCTGCAGCTCGAGTTATTTATTGTTTTTGTTTTTTTTAAC
pGBKT7-Actin(*R.d*)-F	ATGGCCATGGAGGCCGAATTCATGTGTGACGACGAAGTAGC
pGBKT7-Actin(*R.d*)-R	CCGCTGCAGGTCGACGGATCCCTTAGAAGCATTTCCTGTGCAC
pGBKT7-Actin(*N.c*)-F	ATGGCCATGGAGGCCGAATTCATGTGTGACGACGAAGTTGCT
pGBKT7-Actin(*N.c*)-R	CCGCTGCAGGTCGACGGATCCCTTAGAAGCACTTCCTGTGCAC
pGBKT7-Actin(*N.l*)-F	ATGGCCATGGAGGCCGAATTCATGTGTGACGAAGAAGTAGCC
pGBKT7-Actin(*N.l*)-R	CCGCTGCAGGTCGACGGATCCCTTAGAAGCACTTCCTGTGGAC
pMBP-Amp-F	TATCGGAATTAATTCGGATCCGGATGATAAACTAGATTTAAGCAC
pMBP-Amp-R	GTGGTGGTGGTGGTGCTCGAGTTATTTATTGTTTTTGTTTTTTTTAAC

pGEXT-Actin(*R.d*)-F	CCGCGTGGATCCCCGGAATTCATGTGTGACGACGACGTA
pGEXT-Actin(*R.d*)-R	GTCACGATGCGGCCGCTCGAGTTAGAAGCATTTCCTGTGGAC
pGEXT-Actin(*N.c*)-F	CCGCGTGGATCCCCGGAATTCATGTGTGACGACGAAGTTGCT
pGEXT-Actin(*N.c*)-R	GTCACGATGCGGCCGCTCGAGTTAGAAGCACTTCCTGTGCAC
pEF28a(+)-Amp-F	CAGCAAATGGGTCGCGGATCCGATGATAAACTAGATTTA
pEF28a(+)-Amp-R	GCAAGCTTGTCGACGGAGCTCGATGTTTTGTACCAAGGTGTTT
qOsPR1-F	CAGGTGGTGTGGAGCAACTC
qOsPR1-R	GTACGTAGTCCTCCTGCAGC
qOsNPR1-F	TGGACAGGTTATCACCATTGGT
qOsNPR1-R	CCGCAGCTTCCATTCCTATG
qOsPAD4-F	GCCAGCTCCCCTACGACTTC
qOsPAD4-R	CGTGTGCGGTGTAGGTTGTT
qOsACS2-F	CACCCCGAGGCAYCCAT
qOsACS2-R	ATTGGCGATCCTCTTGAACTG
seq-T7-F	TAATACGACTCACTATAGGGC
seq-M13-F	GTTGTAAAACGACGGCCAG
seq-GST-F	GGGCTGGCAAGCCACGTTTGGTG
seq-pMBP-F	ACTGATGAAGGTCTGGAAGCGG
PP2A-F	GACCCTGATGTTGATGTTCGCT
PP2A-R	GAGGGATTTGAAGAGAGATTTC
PVX-Amp-F	TCAGCACCAGCTAGCATCGATATGGATGATAAACTAGATTTAAGCAC
PVX-Amp-R	AACCGTTCATCGGCGGTCGACTTATTTATTGTTTTTGTTTTTTTTAAC
qPVX-CP-F	AAGCCTGAGCACAAATTCGC
qPVX-CP-R	GCTTCAGACGGTGGCCG

qAtRBOHD-F	CTCATTGCCATGCTTCAGTC
qAtRBOHD-R	GTACATAACGAGATTAACGGTCG
qAtCAT2-F	TCAAGGAAGAAGGAGCTTTCA
qAtCAT2-R	CATCATGTGGATCTTCTGTGC
qAtEF1α-F	CAGGCTGATTGTGCTGTTCTTA
qAtEF1α-R	GTTGTATCCGACCTTCTTCAGG
qAtPEN3-F	GTTGAATGAGCTTGCGAGG
qAtPEN3-R	TCAGTGACGAGACTGTTCT

Notes: Homolygous sequences are underlined.
