# Supplementary figures and images for: The Antigenic Membrane Protein (Amp) of Rice Orange Leaf Phytoplasma Suppresses Host Defenses and Is Involved in Pathogenicity
Source: Int J Mol Sci. 2023 Feb 24;24(5):4494. doi: 10.3390/ijms24054494 (PMC10003417; doi:10.3390/ijms24054494)

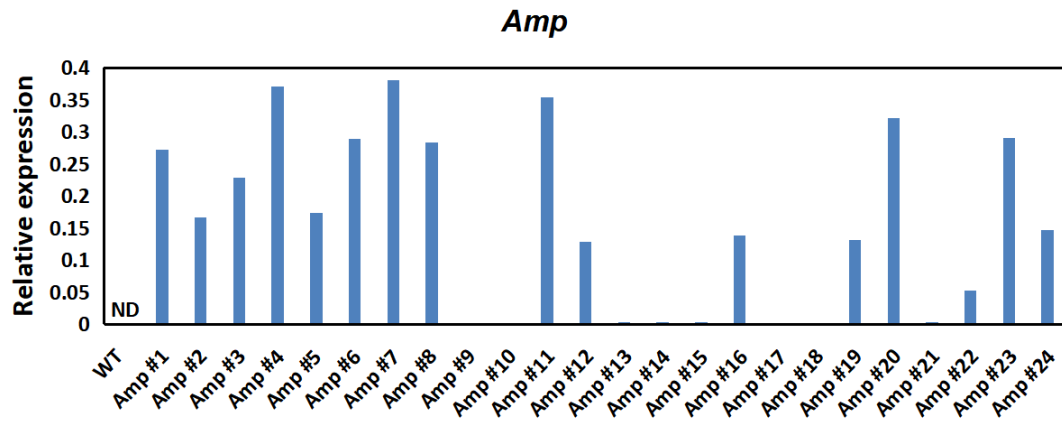

Figure S2: Relative expression of Amp-OE transgenic rice T0 lines.

Supplement: Supplementary file 1 [file ijms-24-04494-s001.zip › Figure S2.pdf]
